# Identification of Three Human POLH Germline Variants Defective in Complementing the UV- and Cisplatin-Sensitivity of POLH-Deficient Cells

**DOI:** 10.3390/ijms24065198

**Published:** 2023-03-08

**Authors:** Mina Yeom, Jin-Kyung Hong, Joo-Ho Shin, Yunjong Lee, Frederick Peter Guengerich, Jeong-Yun Choi

**Affiliations:** 1 Department of Pharmacology, Sungkyunkwan University School of Medicine, Suwon 16419, Republic of Korea; 2 Department of Biochemistry, Vanderbilt University School of Medicine, Nashville, TN 37232-0146, USA

**Keywords:** DNA polymerase η, XPV, germline variant, UV, cisplatin, translesion DNA synthesis

## Abstract

DNA polymerase (pol) η is responsible for error-free translesion DNA synthesis (TLS) opposite ultraviolet light (UV)-induced *cis*-*syn* cyclobutane thymine dimers (CTDs) and cisplatin-induced intrastrand guanine crosslinks. POLH deficiency causes one form of the skin cancer-prone disease xeroderma pigmentosum variant (XPV) and cisplatin sensitivity, but the functional impacts of its germline variants remain unclear. We evaluated the functional properties of eight human POLH germline in silico-predicted deleterious missense variants, using biochemical and cell-based assays. In enzymatic assays, utilizing recombinant pol η (residues 1—432) proteins, the C34W, I147N, and R167Q variants showed 4- to 14-fold and 3- to 5-fold decreases in specificity constants (*k*_cat_/*K*_m_) for dATP insertion opposite the 3’-T and 5′-T of a CTD, respectively, compared to the wild-type, while the other variants displayed 2- to 4-fold increases. A CRISPR/Cas9-mediated POLH knockout increased the sensitivity of human embryonic kidney 293 cells to UV and cisplatin, which was fully reversed by ectopic expression of wild-type pol η, but not by that of an inactive (D115A/E116A) or either of two XPV-pathogenic (R93P and G263V) mutants. Ectopic expression of the C34W, I147N, and R167Q variants, unlike the other variants, did not rescue the UV- and cisplatin-sensitivity in POLH-knockout cells. Our results indicate that the C34W, I147N, and R167Q variants—substantially reduced in TLS activity—failed to rescue the UV- and cisplatin-sensitive phenotype of POLH-deficient cells, which also raises the possibility that such hypoactive germline POLH variants may increase the individual susceptibility to UV irradiation and cisplatin chemotherapy.

## 1. Introduction

Human DNA polymerase (pol) η, a member of Y-family, is involved in translesion DNA synthesis (TLS) for tolerance of DNA damage. Pol η can incorporate nucleotides, with different fidelity, opposite various DNA adducts such as *cis*-*syn* cyclobutane thymine dimers (CTDs), platinum-GpG, *C*^8^-, *N*^2^- and *O*^6^-G adducts, and abasic sites [1,2,3,4,5,6]. In particular, pol η can efficiently catalyze error-free TLS opposite ultraviolet light (UV)-induced *cis*-*syn* CTDs and platinum-induced intrastrand GpG crosslinks, by accommodating these lesions in the active site [7,8].

Inherited pol η deficiency in humans results in a genetic disease, xeroderma pigmentosum variant (XPV), characterized by an increased skin cancer risk and sunlight sensitivity [1]. Diverse pathogenic mutations in the *POLH* gene, including nonsense, frameshift, premature stop, and missense alterations, have been identified in XPV patients [1,9,10,11]. XPV patients suffer from severe adverse effects following cisplatin chemotherapy [12]. XPV fibroblasts are sensitive to UV light and cisplatin but are corrected by transfection of *POLH* cDNA [1,13]. In this context, it is reasonable to postulate that human germline *POLH* variants can alter the TLS activity and thus modify the susceptibility to toxic effects of UV and cisplatin in genetically affected individuals.

To date, a total of ~510 missense germline single nucleotide variants in the human *POLH* gene have been listed in the Ensembl variation database [14], but their functional effects remain uncertain. In silico tools, such as SIFT [15] and Polyphen-2 [16], have been developed to predict the functional effects of missense variants. However, these predictions are not accurate enough to substitute for experimental functional assays, as shown in our previous work on the three other human Y-family pols ι, κ, and REV1 [17,18,19,20]. Therefore, experimental approaches are required to assess the functional effects of unstudied variants to validate the dysfunctional ones.

In this study, we selected eight human germline missense *POLH* variants, positioned in polymerase core domains, and predicted in silico to be deleterious, and then investigated their functional effects using both biochemical and cell-based assays. First, we evaluated catalytic activities of the pol η variants by experiments with primer extension, steady-state kinetics of single nucleotide incorporation, and pol-DNA binding assays using recombinant pol η (1—432) proteins. Next, we confirmed rescue abilities of pol η variants for the UV- and cisplatin-sensitive phenotype of POLH-knockout (KO) cells, by cell-based complementation assays. Here we report that C34W, I147N, and R167Q pol η variants, with considerably diminished activity, could not rescue the POLH-KO cells, while the five other variants—with slightly elevated activity—rescued the cells. These findings are discussed in the context of understanding the potential functional consequences of catalytically altered pol η variants.

## 2. Results

### 2.1. Selection of Human Germline POLH Gene Variants to Study

**Figure 1:**
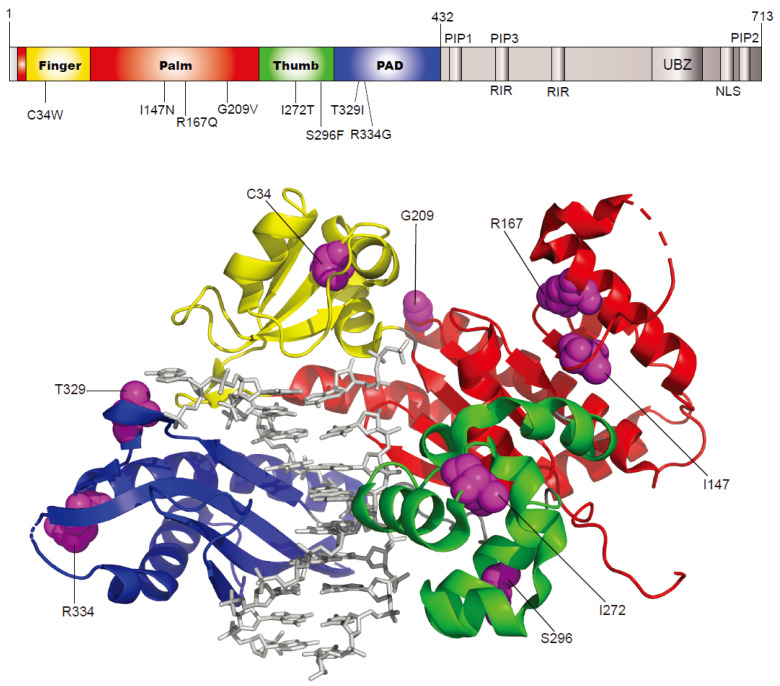
Locations of *POLH* gene variants. The structure (PDB code, 3MR2) of human pol η (1—432) (shown as ribbons) bound to primer/template DNA and incoming nucleotide (shown as gray sticks) is drawn using PyMOL (http://www.pymol.org (accessed on 8 September 2020)). The finger, palm, thumb, and PAD domains are colored yellow, red, green, and blue, respectively. The eight variant residues are indicated in the upper schematic domain diagram and shown as purple spheres in the structure.

We chose eight human germline missense *POLH* variants (Table 1 and Figure 1) that are expected to alter enzyme function on the basis of their location in polymerase core (finger, palm, thumb, and PAD) domains, and deleterious or damaging predictions by SIFT [15] and/or Polyphen-2 [16] from the Ensembl variation database [14].

### 2.2. Effects of Eight POLH Variants on Catalytic Activity of Pol η

**Figure 2:**
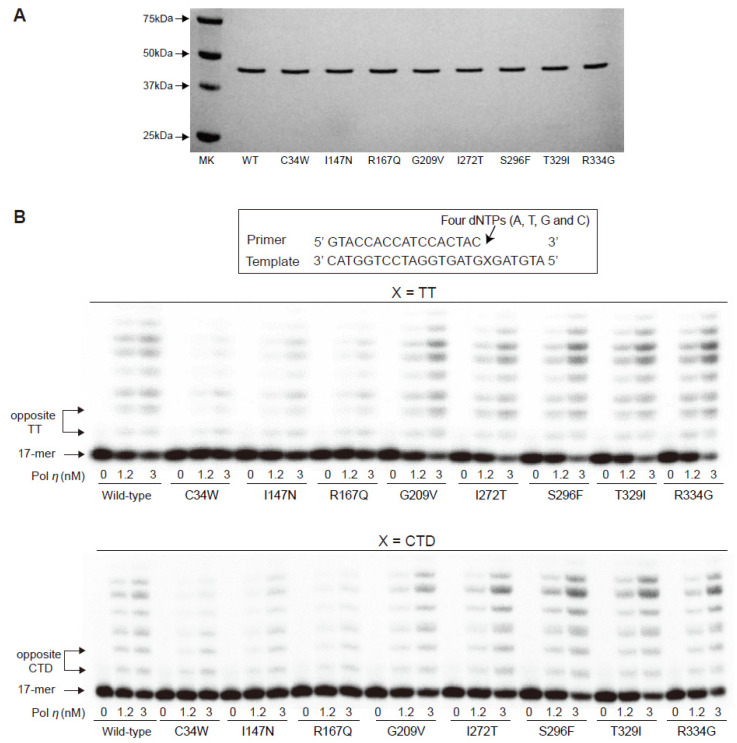
Extension of primers opposite a TT or *cis*-*syn* cyclobutane thymine dimer (CTD) by human pol η (1—432) wild-type and variants. (**A**) Analysis of human pol η (1—432) wild-type and variant proteins by SDS-PAGE. Purified recombinant pol η (1—432) proteins (400 ng each) were separated on a 10% SDS-PAGE gel (*w*/*v*) and visualized by Coomassie brilliant blue staining. Protein size markers are shown on the left. (**B**) The P^32^-labeled 17-mer/25-mer, containing a TT or CTD, was incubated with all four dNTPs (50 μM each) and the indicated concentrations of pol η, for 15 min. The reaction products were analyzed by denaturing PAGE and phosphor imaging. Upper panel: extension opposite TT. Lower panel: extension opposite CTD.

To assess the alterations in catalytic activity of eight pol η variants, we performed “standing-start” primer extensions with wild-type pol η (1—432) and variants, using 17-mer/25-mer duplexes containing a TT or CTD at template position 18–19 from the 3′ end, with all four dNTPs. The C34W, I147N, and R167Q variants generated extension products across the TT or CTD to a substantially lesser extent than the wild-type, while the other five variants yielded slightly more products (Figure 2). These results coincide with the steady-state kinetic data (Table 2). The C34W, I147N, and R167Q variants showed 4- to 14-fold decreases opposite the 3′-T of the CTD and 3- to 5-fold decreases opposite the next 5′-T in *k*_cat_/*K*_m_ (specificity constant, a measure of efficiency) for correct dATP insertion, compared to the wild-type, while the other five variants showed 2- to 4-fold increases in those values. A similar trend of results was observed with unmodified TT templates. The misinsertion frequencies (a measure of fidelity) of eight variants with incorrect dGTP were not very different from those of the wild-type.

### 2.3. Effects of Eight POLH Variants on DNA Substrate Binding of Pol η

To assess the changes in DNA substrate binding affinities of eight pol η variants, we performed fluorescence polarization experiments (Table 3). The *K*_d,DNA_ of each pol η for CTD-containing DNA was similar to that of unmodified DNA, indicating that a CTD placed at the primer–template junction does not affect the DNA-binding affinity of pol η. The *K*_d,DNA_ values of eight variants were not very (≤2-fold) different from the wild-type, indicating that those variants did not considerably alter the DNA-binding affinity of pol η.

### 2.4. Complementation of UV and Cisplatin Sensitivity of POLH-KO Cells by Wild-Type Pol η and Mutants D115A/E116A, R93P, and G263V

**Figure 3:**
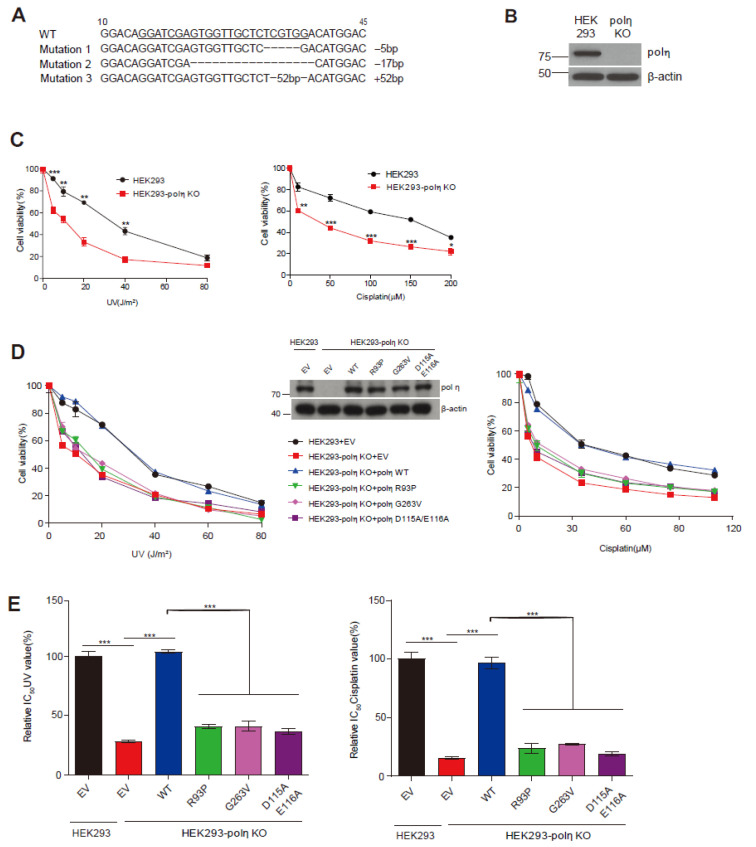
Complementation of UV and cisplatin sensitivity of POLH-knockout (KO) cells with wild-type pol η and D115A/E116A, R93P, and G263V mutants. (**A**) Genomic DNA sequences of the CRISPR/Cas9-mediated POLH-KO HEK293 cell line. The 20-bp target sequence is underlined. Three mutant alleles, which might be related to a near triploid karyotype of HEK 293 cells, with a 5-bp deletion, 17-base deletion, or 52-base addition, resulting in frameshifts at codon 11, 8, or 11, respectively, were verified by DNA sequencing of genomic PCR amplicons from POLH-KO cells. Numbers are relative to the transcription start site. (**B**) Immunoblots showing no pol η expression in POLH-KO cells. (**C**) Increased UV and cisplatin sensitivity of POLH-KO cells. Wild-type and POLH-KO cells were treated with UV radiation (0, 5, 10, 20, 40, or 80 J/m^2^) or cisplatin (0, 10, 50, 100, 150, or 200 μM), and cell viability was determined. Data are shown as means ± SEM from three independent experiments. * *p* < 0.05, ** *p* < 0.01, *** *p* < 0.001 vs. wild-type cells (Student’s *t*-test). (**D**) UV (left panel) and cisplatin (right panel) sensitivity of POLH-KO cells rescued only by wild-type pol η but not by three defective mutants. Wild-type and POLH-KO cells were treated with UV radiation (0, 5, 10, 20, 40, 60, or 80 J/m^2^) or cisplatin (0, 10, 35, 60, 85, or 110 μM), and cell viability was determined. Middle panel: Representative immunoblots of cell lysates (20 μg) from wild-type and POLH-KO cells transfected with the indicated vectors. EV, empty vector. (**E**) Relative IC_50_ UV (left panel) and IC_50_ cisplatin (right panel) values for wild-type cells and POLH-KO cells expressing wild-type or mutants. IC_50_ values calculated from Figure 3D were normalized to wild-type cells. Data are shown as mean ± SEM from three independent experiments. *** *p* < 0.001 (ANOVA with Tukey’s test).

We developed POLH-KO cell-based complementation assays to evaluate the capability of each pol η variant to rescue the UV- and cisplatin-sensitive phenotype in POLH-KO cells. First, the POLH-KO HEK293 cell line was generated using a CRISPR/Cas9 system, and verified at the gene and protein level (Figure 3A,B). Second, we confirmed the distinct phenotype of POLH-KO cells, i.e., the enhanced sensitivity to UV and cisplatin, compared to wild-type cells, by CCK8 cell viability assays (Figure 3C), as similarly reported earlier with XPV fibroblasts [1,13,21]. This phenotype was readily discernible in our assay condition without caffeine. Third, to validate this assay, we confirmed that the ectopic expression of wild-type pol η can reverse the UV- and cisplatin-sensitivity of POLH-KO cells to the wild-type cell level but, in sharp contrast, that of a catalytically inactive D115A/E116A mutant [22], and two known XPV-pathogenic defective mutants R93P and G263V [9,10], could not reverse the sensitivity (Figure 3D), indicating that the catalytically intact pol η is required for the resistance of cells to UV and cisplatin. These features were also clearly demonstrated by comparison of the relative IC_50_ values of UV and cisplatin (Figure 3E), which were useful as indicators of cell sensitivity to genotoxic agents in cell-based assays with POLI-KO HEK293 cells [18]. Ectopic expression of wild-type pol η, but not that of three defective mutants, reversed both IC_50_ values of the POLH-KO cells treated with UV and cisplatin to the wild-type cell level. In this assay, the protein levels of ectopically expressed pol η were similar to the endogenous level of HEK293 cells (Figure 3D, middle).

### 2.5. Capabilities of Eight POLH Variants to Rescue the UV- and Cisplatin-Sensitivity of POLH-KO Cells

**Figure 4:**
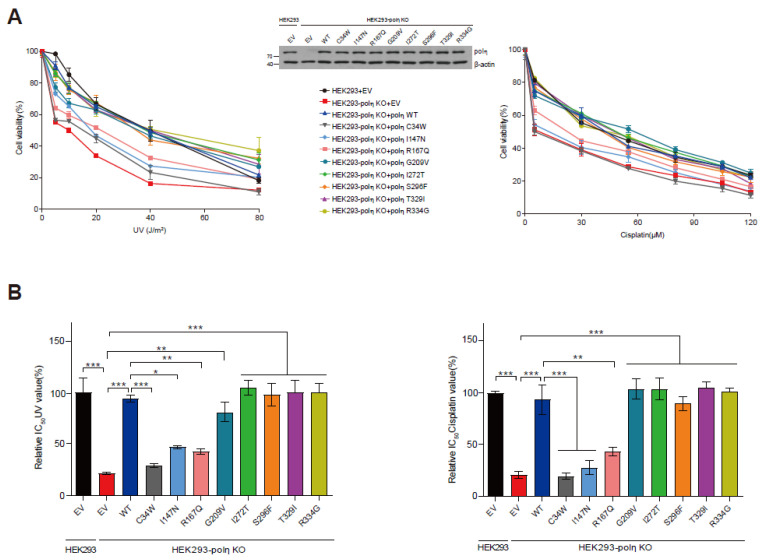
Effects of eight POLH variants on UV and cisplatin sensitivity in POLH-KO cells. (**A**) Abilities of eight pol η variants to rescue UV (left panel) and cisplatin (right panel) sensitivity in POLH-KO cells. Wild-type and POLH-KO cells transfected with indicated vectors were treated with UV radiation (0, 5, 10, 20, 40, and 80 J/m^2^) or cisplatin (0, 5, 30, 55, 80, 105, and 120 μM), and cell viability was determined. Middle panel: Representative immunoblots of cell lysates (20 μg) from wild-type and POLH-KO cells transfected with the indicated vectors. (**B**) Relative IC_50_ UV (left panel) and IC_50_ cisplatin (right panel) values for wild-type cells and POLH-KO cells expressing wild-type or variants. IC_50_ values calculated from Figure 4A were normalized to wild-type cells. Data are shown as means ± SEM from three independent experiments. * *p* < 0.05, ** *p* < 0.01, *** *p* < 0.001 (ANOVA with Tukey’s test).

We employed this POLH-KO cell complementation assay to evaluate the capability of each pol η variant to rescue the UV- and cisplatin-sensitivity of POLH-KO cells. The C34W, I147N, and R167Q variants did not rescue the UV- and cisplatin-sensitivity (Figure 4A), with no significant improvements in either the IC_50_ UV or cisplatin values (Figure 4B). In contrast, the other five variants fully rescued the UV- and cisplatin-sensitivity of POLH-KO cells (Figure 4A), with significant improvements in their relative IC_50_ UV and cisplatin values, nearly to the wild-type cell level (Figure 4B). Under this assay condition, the protein levels of ectopically expressed pol η were similar to the endogenous level of HEK293 cells (Figure 4A middle).

## 3. Discussion

In this study, we evaluated the functional properties of eight germline in silico-predicted deleterious missense variants of human pol η, at the molecular and cellular level, and identified three hypoactive variants as defective in rescuing the UV- and cisplatin-sensitivity of POLH-deficient cells. Together with enzymatic analyses, we employed cell-based assays to assess the complementation ability of each variant, based on the capability of the ectopically expressed *POLH* gene (but not the defective mutant genes) to rescue the UV- and cisplatin-sensitive phenotype of POLH-KO cells (Figure 3). Here we report that the C34W, I147N, and R167Q variants, which were respectively 74-, 35-, and 10-fold reduced in the overall TLS efficiency (i.e., *k*_cat_/*K*_m_ for dATP insertion opposite 3′-T× that opposite 5′-T, Table 2) opposite a CTD, failed to rescue the POLH-KO cells from the enhanced sensitivity to UV and cisplatin (Figure 4), suggesting that at least such hypoactive (≥10-fold reduction in the overall TLS efficiency) pol η variants might not be sufficient to protect cells from UV and cisplatin.

This is the first report, to our knowledge, to identify the functionally defective germline missense variants of human *POLH* gene in a “bottom-up” approach from the Ensembl variation database [14], without using the XPV patient samples, in which in silico-predicted deleterious variants are screened biochemically and then confirmed by cell-based assays. This strategy was also successfully applied to identify dysfunctional POLI variants in our previous work [18]. Among eight variants that are predicted to be deleterious by the SIFT and/or PolyPhen-2 algorithms, only three variants were found to be dysfunctional in our assays. Although not perfect, the PolyPhen-2 algorithm (based on both sequence and structural features) [16] appeared to show a higher percentage (60%) of correct prediction of deleterious variants than the sequence homology-based SIFT algorithm (38%) [15]. We also note that all eight variants had combined annotation dependent depletion (CADD) scores (ranging from 22 to 29) higher than a possible cutoff value of 20, indicating that they are predicted to be among the top 1% most deleterious variants in the human genome by the CADD algorithm, integrating many diverse functional annotations [23]. Such discordance between functional assay results and in silico prediction effects is also observed with the TP53 and BRCA1/2 missense variants [24,25]. These observations highlight the necessity of experimental assays to validate the possibly deleterious genetic variants predicted by in silico tools.

The eight studied POLH variants can be divided into two types, according to the rescue capability (Figure 4). The first type is the functionally defective ones (C34W, I147N, and R167Q), which were incapable of rescuing POLH-KO cells, with substantial impairments in catalytic activity. Noticeably, the C34W variant caused a severe catalytic impairment. It is plausible that the substitution to a bulky hydrophobic Trp at the beginning of the β2-strand in the finger domain would perturb the conformation of the β2-strand, at the end of which Gln-38 stabilizes the nascent base pair by hydrogen bonding with the template base [7], and thus destabilize the pol η active site. Interestingly, the moderately hypoactive R167Q variant was also deprived of the rescue ability, implying a demand of a certain minimum activity of pol η for providing tolerance to UV and cisplatin in cells. However, there also exists the possibility that these hypoactive variants are still at least partially functional in certain tissue or cell types, because their actual outcomes likely depend on the stress conditions and the levels of pol η and other TLS polymerases, which may vary with tissue or cell type. The other five variants belong to the functionally competent type, which could fully rescue POLH-KO cells, albeit with slightly increased TLS activities. The I272T variant, observed in several melanomas [26], was revealed to be functionally competent. Interestingly, all these slightly hyperactive variants did not show “over-rescue” (i.e., greater than wild-type) effects, which was similarly observed with three pol ι variants that were slightly hyperactive against H_2_O_2_ sensitivity [18]. This finding agrees with the earlier observation, that a large overexpression of pol η (~59-fold above the endogenous mRNA level of human fibroblasts) restores the UV cytotoxicity of XPV fibroblasts nearly to the range obtained with normal fibroblasts [27]. These observations suggest that the protective effect of pol η against UV and cisplatin, is likely saturated at endogenous levels of pol η in those cells. It might also be attributed to the finding that the actual functioning of pol η is tightly regulated by multiple post-translational mechanisms including phosphorylation, ubiquitination, and PCNA monoubiquitination in cells [28,29,30].

In conclusion, our results suggest that three human germline *POLH* variants may substantially impair the TLS activity of pol η and thus lead to deprivation of its protective function against UV and cisplatin stresses in cells, which might possibly serve as predisposing factors for individual susceptibility to UV radiation and cisplatin chemotherapy. Although not conclusive yet, a genetically hypoactive status of pol η might potentially increase a cancer risk in humans, in that heterozygous POLH-deficient mice show an increased incidence of UV-induced skin cancer [31]. Our POLH-KO cell-based functional assays seem to be fairly quick and easy, and thus would also be useful for initial screening of unstudied non-synonymous POLH variants, in advance of the biochemical assays that reveal the mechanistic details. The exact clinical implications of the human germline dysfunctional POLH variants remain unclear and further evaluation of in vivo outcomes of these and other undetermined POLH variants would allow a better understanding of the role of POLH variants in interindividual variability in cancer risks and platinum drug responses.

## 4. Materials and Methods

### 4.1. DNA Substrates

The 25-mer (3′−CATGGTGGTAGGTGATGXGATGTA−5′; X = TT or CTD), 17-mer (5′−GTACCACCATCCACTAC−3′), 18-mer (5′−GTACCACCATCCACTACA−3′), and 13-FAM-mer (5′−(FAM)-CACCATCCACTAC−3′; FAM = 6-carboxyfluorescein) oligonucleotides were obtained from Bioneer (Daejeon, Korea). DNA substrates were prepared as described [19]. 5′-^32^P-labeled 17-mers (or 18-mers) and 13-FAM-mers were annealed with 25-mer templates, respectively, for polymerase activity and pol-DNA binding assays.

### 4.2. Expression Vector Construction for Pol η (1—432) Variants and Protein Purification

DNAs encoding the pol η core (residues 1—432) with a C-terminal 6×His tag, were synthesized using *Escherichia coli*-optimized codons from GenScript (Piscataway, NJ, USA) and cloned into the *Nco*I and *Xho*I restriction sites of a pET28a vector. Each mutation for the eight variants was created in the vector by PCR-based site-directed mutagenesis, using a QuickChange mutagenesis kit (Agilent, Santa Clara, CA, USA). The oligonucleotide primers for introducing the point mutation were 5′−CTCATTTGAGGAATAAACCTTGGGCAGTTGTACAGTACAAATCAT−3′ for C34W, 5′−AGACTTGTTGCCAAGCACTTACAATGAAGGGTTGCC−3′ for I147N, 5′−CTGTTCAGAAAGAGGGGATGCAAAAACAAGGCTTATTTCAATG−3′ for R167Q, 5′−CATAGAGAGGGAGACTGTTTTTCAGTGTTCAGCTG−3′ for G209V, 5′−TGTCATTGAGATCCTAGGGACGGAATACATGGGTGAACTGAC−3′ for I272T, 5′−TGGGGAGAAGAATGGGTTTTGGCTATATGCCATGT−3′ for S296F, 5′−GCTGTAGTAAGAACTTCCCAGGAAAAATAGCTCTTGCTACTC−3′ for T329I, 5′−CAGGAAAAACAGCTCTTGCTACTGGGGAACAGGTAC−3′ for R334G, and the corresponding antiparallel primer for each mutation. All substitutions were confirmed by DNA sequencing. Recombinant His-tagged pol η core was expressed in *E. coli* strain BL21 (DE3) cells harboring each vector, in Terrific Broth containing kanamycin (50 μg mL^−1^), by induction with 0.2 mM isopropyl-*β*-D-1-thiogalactopyranoside, at an OD_600_ of 0.6 and incubation overnight at 26 °C. The cell pellets were lysed and centrifuged, and the resulting supernatant was subjected to sequential chromatography on a 1 mL HisTrap column and a Mono-S column (GE Healthcare, Piscataway, NJ, USA), as described previously [19]. Pol η core was eluted at ~500 mM NaCl. The homogeneity of purified proteins was confirmed by SDS polyacrylamide gel electrophoresis (PAGE) and Coomassie brilliant blue staining (Figure 2A).

### 4.3. Enzyme Assays and Steady-State Kinetic Analysis

DNA polymerase reactions and steady-state kinetic analyses were performed as described previously [5]. The reactions contained 50 mM Tris-HCl (pH 7.5), 5 mM dithiothreitol, 100 μg mL^−1^ bovine serum albumin (*w*/*v*), 10% glycerol (*v*/*v*), 5 mM MgCl_2_, and 100 nM DNA substrates (i.e., 5′-^32^P-labeled 17-mer (or 18-mer) primers annealed to 25-mer templates containing a TT or CTD). Reactions were started by the addition of dNTPs and MgCl_2_ to preincubated polymerase/DNA mixtures and ended with six volumes of a solution of 20 mM EDTA in 95% formamide (*v*/*v*). For steady-state kinetic analysis, the primer-template was extended in the presence of 0.4−1 nM pol η, with increasing concentrations of individual dNTPs, for 10 min, where the maximal product formation was ≤20% of the substrate concentration. Products were separated by 8 M urea-16% PAGE and analyzed with a PMI system (Bio-Rad, Hercules, CA, USA), as described previously [5]. Graphs of the product formation rates versus dNTP concentration were fit to the Michaelis−Menten equation in GraphPad Prism 7.0 (GraphPad Software, San Diego, CA, USA), for the determination of *k*_cat_ and *K*_m_ values.

### 4.4. Fluorescence Polarization

The 13-FAM-mer/25-mer (2 nM) was incubated with varying concentrations of pol η. The binding reactions contained 50 mM HEPES-KOH (pH 7.5), 10 mM potassium acetate, 2 mM β-mercaptoethanol, 0.1 mg/mL^−1^ BSA, and 5 mM MgCl_2_. Fluorescence polarization was measured with a Synergy Neo plate reader (Biotek, Winooski, VT, USA), using 485 and 528 nm excitation and emission filters, respectively, and *K*_d,DNA_ (equilibrium dissociation constant for DNA binding) values were estimated as described previously [19]. The fluorescence polarization data (as a function of enzyme concentration) were plotted to estimate *K*_d,DNA_ by fitting to a quadratic equation: *P = P*_0_ *+* (*P*_max_ *− P*_0_)((*D*_t_ *+ E*_t_ *+ K*_d,DNA_) − ((*D*_t_ *+ E*_t_ *+ K*_d,DNA_)^2^ − (4*D*_t_*E*_t_))^1/2^)/(2*D*_t_), where *P* is the measured change in polarization (in units of millipolarization), *P*_0_ is the initial polarization (DNA alone), *P*_max_ is the maximum polarization, *D*_t_ is the total DNA concentration, and *E*_t_ is the total enzyme concentration, using the GraphPad Prism 7.0 software.

### 4.5. Mammalian Expression Vector Construction, Cell Culture, and Transfection

The POLH coding cDNA [5] was cloned into the *BamH*I and *Xho*I restriction sites of a pcDNA3.1(+) vector. Each mutation, for eight variants and three mutants, was introduced into the vector by site-directed mutagenesis (vide supra, Section 4.2). The mutagenic oligonucleotide primers were 5′−CGCAACAAACCGTGGGCCGTGGTTCAGTA−3′ for C34W, 5′−GCTGCCGTCCACCTATAACGAAGGTCTGC−3′ for I147N, 5′−GGTGCAAAAAGAAGGTATGCAGAAACAGGGCCTGTTTCAATG−3′ for R167Q, 5′−ATCGAACGCGAAACCGTTTTTCAGTGCTCAGCG−3′ for G209V, 5′− CTCCGTTATTGAAATCCTGGGTACTGAATATATGGGCGA−3′ for I272T, 5′−CATTTCGGCGAGAAAAACGGCTTCTGGCTGTACGCAATGT−3′ for S296F, 5′−TGTTCTAAAAATTTTCCGGGTAAAATCGCACTGGCAACG−3′ for T329I, 5′−GCACTGGCAACGGGCGAACAGGTCC−3′ for R334G, 5′−CCTCACCAAGTACCCGGAAGCCAGTGTTG−3′ for R93P, 5′−CTTGGAGGAAAGCTAGTGGCCTCTGTCATTGAG−3′ for G263V, 5′−ATTGAACGTGCCAGCATTGCTGCGGCTTACGTAGATCTGAC−3′ for D115A/E116A, and the corresponding antiparallel primers. All substitutions were confirmed by DNA sequencing. Human embryonic kidney (HEK) 293 cells (Korean Cell Line Bank, Seoul, Republic of Korea) were cultured at 37 °C in a 5% CO_2_ (*v*/*v*) atmosphere, in Dulbecco’s modified eagle medium with 10% (*v*/*v*) fetal bovine serum. Cells were seeded on 6-well plates, at a cell density of 1 × 10^6^ cells/well, incubated overnight, and transfected with the expression vector (0.5 μg) using Lipofectamine 3000 (Thermo Fisher, Waltham, MA, USA), following the manufacturer’s instructions. Forty-eight hours after transfection, the cells were collected and used for the subsequent assays.

### 4.6. POLH-KO Cell Line Generation and Immunoblotting

The CRISPR/Cas9-mediated POLH-KO HEK293 cell line was generated as described previously [32]. The guide RNA (5′−CACCGGGATCGAGTGGTTGCTCTCG−3′) targeting the exon 1 was designed using the CRISPR design tool (http://crispr.mit.edu (accessed on 5 October 2018)). Cells were transduced with gRNA-encoding lentiviruses generated from LentiCRISPRV2 (Addgene #52961). Infected cells were selected using puromycin (2 μg mL^−1^), and single cell clones were obtained through limited dilution in 96-well plates. The POLH knockout was confirmed by immunoblotting and genomic sequencing from candidate clones. Cell lysate preparation and immunoblotting was performed as described in [18], using anti-pol η (A301-231A, Bethyl Laboratories, Montgomery, TX, USA), anti-β-actin (GTX629630, Genetex, Irvine, CA, USA), anti-mouse IgG (GTX213111-01, Genetex), and anti-rabbit IgG (GTX213110-01, Genetex) antibodies.

### 4.7. Cell Viability Assay

Cells were seeded at 1.0 × 10^4^ cells/well on 96-well plates, cultured overnight, and exposed to UV radiation or cisplatin (for 48 h) at varying doses. For UV radiation, cells were resuspended in PBS buffer, exposed to UV (254 nm) using a CL-1000 crosslinker (UVP, Upland, CA, USA), and incubated with fresh medium for 24 h. After treatment, cell viability was measured using CCK-8 (CK04; Dojindo, Kumamoto, Japan) following the manufacturer’s instructions.

### 4.8. Statistical Analysis

Statistical comparisons were performed using Student’s *t*-test or one-way analysis of variance (ANOVA) with Tukey’s multiple comparison test. *p* < 0.05 was considered statistically significant.

## Figures and Tables

**Table 1:** Human germline POLH variants studied.

rs ID ^a^	Nucleotide Change	Amino Acid Change	Protein Domain	Minor Allele Frequency ^b^			In Silico Prediction	
				1000 Genomes	ESP	gnomAD	SIFT	PolyPhen-2
rs371810027	c.102T > G	C34W	finger	-	0.00008	-	deleterious	probably damaging
rs200366966	c.440T > A	I147N	palm	0.0002	-	0.00000398	deleterious	probably damaging
rs201365711	c.500G > A	R167Q	palm	0.0002	-	0.0000247	deleterious	possibly damaging
rs2307456	c.626G > T	G209V	palm	0.0042	0.00223	0.00364	deleterious	probably damaging
rs147712217	c.815T > C	I272T	thumb	0.0008	0.00054	0.00106	deleterious	benign
rs200149644	c.887C > T	S296F	thumb	-	-	0.000163	deleterious	probably damaging
rs35675573	c.986C > T	T329I	PAD	0.0046	0.00546	0.00174	deleterious	benign
rs9333548	c.1000C > G	R334G	PAD	0.0014	0.00177	0.000605	deleterious	benign

^a^ Reference SNP identification number. ^b^ From the 1000 genomes project, the NHLBI exome sequencing project (ESP), and the genome aggregation database (gnomAD, v2.1.1).

**Table 2:** Steady-state kinetic parameters for dATP incorporation opposite the 3′- and 5′-T of a TT or CTD by human wild-type pol η (1—432) and variants.

DNA Template	Template Base	Pol η (1—432)	*K*_m_ (μM)	*k*_cat_ (s^−1^)	*k*_cat_/*K*_m_ (s^−1^ μM^−1^)	Relative Efficiency ^a^	*f*_mis_ ^b^
TT	3′-T	wild-type	0.38 ± 0.04	0.076 ± 0.002	0.20 ± 0.02	1	0.17
		C34W	1.3 ± 0.2	0.032 ± 0.004	0.025 ± 0.003	0.13	0.056
		I147N	0.80 ± 0.06	0.0096 ± 0.0002	0.012 ± 0.001	0.06	0.36
		R167Q	0.26 ± 0.04	0.018 ± 0.001	0.069 ± 0.010	0.35	0.14
		G209V	0.20 ± 0.05	0.023 ± 0.002	0.12 ± 0.01	0.60	0.27
		I272T	0.081 ± 0.019	0.041 ± 0.002	0.51 ± 0.10	2.6	0.084
		S296F	0.12 ± 0.02	0.052 ± 0.002	0.43 ± 0.06	2.2	0.10
		T329I	0.17 ± 0.03	0.051 ± 0.003	0.30 ± 0.05	1.5	0.16
		R334G	0.15 ± 0.04	0.073 ± 0.007	0.49 ± 0.14	2.5	0.12
	5′-T	wild-type	0.087 ± 0.017	0.096 ± 0.005	1.1 ± 0.2	1	0.17
		C34W	0.11 ± 0.01	0.030 ± 0.001	0.27 ± 0.02	0.25	0.056
		I147N	0.084 ± 0.008	0.021 ± 0.001	0.25 ± 0.02	0.23	0.11
		R167Q	0.074 ± 0.010	0.032 ± 0.001	0.43 ± 0.05	0.39	0.095
		G209V	0.048 ± 0.010	0.053 ± 0.002	1.1 ± 0.2	1.0	0.21
		I272T	0.011 ± 0.001	0.029 ± 0.001	2.6 ± 0.2	2.4	0.24
		S296F	0.04 ± 0.01	0.056 ± 0.002	1.4 ± 0.2	1.3	0.41
		T329I	0.011 ± 0.001	0.032 ± 0.001	2.9 ± 0.3	2.6	0.13
		R334G	0.016 ± 0.002	0.038 ± 0.002	2.4 ± 0.3	2.2	0.20
CTD	3′-T	wild-type	0.39 ± 0.05	0.066 ± 0.003	0.17 ± 0.02	1	0.14
		C34W	0.72 ± 0.07	0.0083 ± 0.0002	0.012 ± 0.001	0.07	0.066
		I147N	0.42 ± 0.07	0.0075 ± 0.0003	0.018 ± 0.002	0.11	0.24
		R167Q	0.48 ± 0.05	0.023 ± 0.001	0.048 ± 0.005	0.28	0.085
		G209V	0.23 ± 0.05	0.061 ± 0.005	0.27 ± 0.06	1.6	0.063
		I272T	0.025 ± 0.007	0.017 ± 0.001	0.68 ± 0.16	4.0	0.050
		S296F	0.11 ± 0.01	0.034 ± 0.001	0.31 ± 0.02	1.8	0.11
		T329I	0.054 ± 0.009	0.038 ± 0.002	0.70 ± 0.11	4.1	0.031
		R334G	0.13 ± 0.02	0.077 ± 0.006	0.59 ± 0.09	3.5	0.049
	5′-T	wild-type	0.065 ± 0.005	0.044 ± 0.005	0.68 ± 0.04	1	0.053
		C34W	0.10 ± 0.02	0.013 ±0.001	0.13 ± 0.02	0.19	0.016
		I147N	0.060 ± 0.006	0.011 ± 0.0003	0.18 ± 0.01	0.26	0.018
		R167Q	0.11 ± 0.01	0.028 ± 0.001	0.25 ± 0.02	0.37	0.052
		G209V	0.030 ± 0.003	0.032 ± 0.001	1.1 ± 0.1	1.6	0.032
		I272T	0.0081 ± 0.0008	0.015 ± 0.0004	1.9 ± 0.2	2.8	0.022
		S296F	0.017 ± 0.003	0.030 ± 0.001	1.8 ± 0.3	2.6	0.037
		T329I	0.013 ± 0.001	0.020 ± 0.001	1.5 ± 0.1	2.2	0.080
		R334G	0.020 ± 0.002	0.023 ± 0.001	1.2 ± 0.1	1.8	0.067

^a^ Relative efficiency, calculated by dividing *k*_cat_/*K*_m_ of each variant for dATP insertion opposite T, by *k*_cat_/*K*_m_ of wild-type pol η. ^b^ Misinsertion frequency, calculated by dividing *k*_cat_/*K*_m_ for dGTP misinsertion by *k*_cat_/*K*_m_, for dATP insertion opposite T.

**Table 3:** DNA binding affinities of human wild-type pol η (1—432) and variants.

pol η (1—432)	*K*_d_ (nM)	
	13-FAM-mer/25-TT-mer	13-FAM-mer/25-CTD-mer
wild-type	10 ± 1	11 ± 2
C34W	6.1 ± 0.6	5.9 ± 0.8
I147N	6.7 ± 1.2	7.0 ± 1.5
R167Q	9.5 ± 1.2	18 ± 3
G209V	8.4 ± 1.0	11 ± 2
I272T	8.5 ± 1.9	11 ± 2
S296F	10 ± 2	14 ± 2
T329I	5.0 ± 0.9	5.9 ± 0.7
R334G	11 ± 2	12 ± 2

## Data Availability

Not applicable.

## Abbreviations

CTD	*cis*-*syn* cyclobutane thymine dimer
EV	empty vector
IC_50_	concentration that induces 50% inhibition of cell viability
KO	knockout
TLS	translesion DNA synthesis
UV	ultraviolet light
WT	wild-type

## References

[1] Masutani C., Kusumoto R., Yamada A., Dohmae N., Yokoi M., Yuasa M., Araki M., Iwai S., Takio K., Hanaoka F. (1999). The *XPV* (xeroderma pigmentosum variant) gene encodes human DNA polymerase η. Nature.

[2] Vaisman A., Masutani C., Hanaoka F., Chaney S.G. (2000). Efficient translesion replication past oxaliplatin and cisplatin GpG adducts by human DNA polymerase η. Biochemistry.

[3] Choi J.-Y., Stover J.S., Angel K.C., Chowdhury G., Rizzo C.J., Guengerich F.P. (2006). Biochemical basis of genotoxicity of heterocyclic arylamine food mutagens: Human DNA polymerase η selectively produces a two-base deletion in copying the *N*^2^-guanyl adduct of 2-amino-3-methylimidazo[4,5-*f*]quinoline but not the *C*^8^ adduct at the NarI G3 site. J. Biol. Chem..

[4] Choi J.-Y., Chowdhury G., Zang H., Angel K.C., Vu C.C., Peterson L.A., Guengerich F.P. (2006). Translesion synthesis across *O*^6^-alkylguanine DNA adducts by recombinant human DNA polymerases. J. Biol. Chem..

[5] Choi J.-Y., Guengerich F.P. (2005). Adduct size limits efficient and error-free bypass across bulky *N*^2^-guanine DNA lesions by human DNA polymerase η. J. Mol. Biol..

[6] Choi J.-Y., Lim S., Kim E.J., Jo A., Guengerich F.P. (2010). Translesion synthesis across abasic lesions by human B-family and Y-family DNA polymerases α, δ, η, ι, κ, and REV1. J. Mol. Biol..

[7] Biertumpfel C., Zhao Y., Kondo Y., Ramon-Maiques S., Gregory M., Lee J.Y., Masutani C., Lehmann A.R., Hanaoka F., Yang W. (2010). Structure and mechanism of human DNA polymerase η. Nature.

[8] Zhao Y., Biertumpfel C., Gregory M.T., Hua Y.J., Hanaoka F., Yang W. (2012). Structural basis of human DNA polymerase η-mediated chemoresistance to cisplatin. Proc. Natl. Acad. Sci. USA.

[9] Broughton B.C., Cordonnier A., Kleijer W.J., Jaspers N.G., Fawcett H., Raams A., Garritsen V.H., Stary A., Avril M.F., Boudsocq F. (2002). Molecular analysis of mutations in DNA polymerase η in xeroderma pigmentosum-variant patients. Proc. Natl. Acad. Sci. USA.

[10] Opletalova K., Bourillon A., Yang W., Pouvelle C., Armier J., Despras E., Ludovic M., Mateus C., Robert C., Kannouche P. (2014). Correlation of phenotype/genotype in a cohort of 23 xeroderma pigmentosum-variant patients reveals 12 new disease-causing *POLH* mutations. Hum. Mutat..

[11] Feltes B.C., Menck C.F.M. (2022). Current state of knowledge of human DNA polymerase eta protein structure and disease-causing mutations. Mutat. Res. Rev. Mutat. Res..

[12] Sumiyoshi M., Soda H., Sadanaga N., Taniguchi H., Ikeda T., Maruta H., Dotsu Y., Ogawara D., Fukuda Y., Mukae H. (2017). Alert Regarding Cisplatin-induced Severe Adverse Events in Cancer Patients with Xeroderma Pigmentosum. Intern. Med..

[13] Yamada A., Masutani C., Iwai S., Hanaoka F. (2000). Complementation of defective translesion synthesis and UV light sensitivity in xeroderma pigmentosum variant cells by human and mouse DNA polymerase η. Nucleic Acids Res..

[14] Hunt S.E., McLaren W., Gil L., Thormann A., Schuilenburg H., Sheppard D., Parton A., Armean I.M., Trevanion S.J., Flicek P. (2018). Ensembl variation resources. Database.

[15] Ng P.C., Henikoff S. (2001). Predicting deleterious amino acid substitutions. Genome Res..

[16] Adzhubei I.A., Schmidt S., Peshkin L., Ramensky V.E., Gerasimova A., Bork P., Kondrashov A.S., Sunyaev S.R. (2010). A method and server for predicting damaging missense mutations. Nat. Methods.

[17] Kim J., Song I., Jo A., Shin J.-H., Cho H., Eoff R.L., Guengerich F.P., Choi J.-Y. (2014). Biochemical analysis of six genetic variants of error-prone human DNA polymerase ι involved in translesion DNA synthesis. Chem. Res. Toxicol..

[18] Yeom M., Hong J.K., Kim J.K., Guengerich F.P., Choi J.-Y. (2020). Three human pol ι variants with impaired polymerase activity fail to rescue H_2_O_2_ sensitivity in *POLI*-deficient cells. Chem. Res. Toxicol..

[19] Kim J.-K., Yeom M., Hong J.-K., Song I., Lee Y.-S., Guengerich F.P., Choi J.-Y. (2016). Six germline genetic variations impair the translesion synthesis activity of human DNA polymerase κ. Chem. Res. Toxicol..

[20] Yeom M., Kim I.-H., Kim J.-K., Kang K., Eoff R.L., Guengerich F.P., Choi J.-Y. (2016). Effects of twelve germline missense variations on DNA lesion and G-quadruplex bypass activities of human DNA polymerase REV1. Chem. Res. Toxicol..

[21] Bassett E., King N.M., Bryant M.F., Hector S., Pendyala L., Chaney S.G., Cordeiro-Stone M. (2004). The role of DNA polymerase η in translesion synthesis past platinum-DNA adducts in human fibroblasts. Cancer Res..

[22] Rey L., Sidorova J.M., Puget N., Boudsocq F., Biard D.S., Monnat R.J., Cazaux C., Hoffmann J.S. (2009). Human DNA polymerase η is required for common fragile site stability during unperturbed DNA replication. Mol. Cell. Biol..

[23] Rentzsch P., Witten D., Cooper G.M., Shendure J., Kircher M. (2019). CADD: Predicting the deleteriousness of variants throughout the human genome. Nucleic Acids Res..

[24] Miosge L.A., Field M.A., Sontani Y., Cho V., Johnson S., Palkova A., Balakishnan B., Liang R., Zhang Y., Lyon S. (2015). Comparison of predicted and actual consequences of missense mutations. Proc. Natl. Acad. Sci. USA.

[25] Sadowski C.E., Kohlstedt D., Meisel C., Keller K., Becker K., Mackenroth L., Rump A., Schrock E., Wimberger P., Kast K. (2017). *BRCA1/2* missense mutations and the value of in-silico analyses. Eur. J. Med. Genet..

[26] Di Lucca J., Guedj M., Lacapere J.J., Fargnoli M.C., Bourillon A., Dieude P., Dupin N., Wolkenstein P., Aegerter P., Saiag P. (2009). Variants of the xeroderma pigmentosum variant gene (*POLH*) are associated with melanoma risk. Eur. J. Cancer.

[27] King N.M., Nikolaishvili-Feinberg N., Bryant M.F., Luche D.D., Heffernan T.P., Simpson D.A., Hanaoka F., Kaufmann W.K., Cordeiro-Stone M. (2005). Overproduction of DNA polymerase η does not raise the spontaneous mutation rate in diploid human fibroblasts. DNA Repair.

[28] Chen Y.W., Cleaver J.E., Hatahet Z., Honkanen R.E., Chang J.Y., Yen Y., Chou K.M. (2008). Human DNA polymerase η activity and translocation is regulated by phosphorylation. Proc. Natl. Acad. Sci. USA.

[29] Bienko M., Green C.M., Sabbioneda S., Crosetto N., Matic I., Hibbert R.G., Begovic T., Niimi A., Mann M., Lehmann A.R. (2010). Regulation of translesion synthesis DNA polymerase η by monoubiquitination. Mol. Cell.

[30] Kannouche P.L., Wing J., Lehmann A.R. (2004). Interaction of human DNA polymerase η with monoubiquitinated PCNA: A possible mechanism for the polymerase switch in response to DNA damage. Mol. Cell.

[31] Lin Q., Clark A.B., McCulloch S.D., Yuan T., Bronson R.T., Kunkel T.A., Kucherlapati R. (2006). Increased susceptibility to UV-induced skin carcinogenesis in polymerase η-deficient mice. Cancer Res..

[32] Shalem O., Sanjana N.E., Hartenian E., Shi X., Scott D.A., Mikkelson T., Heckl D., Ebert B.L., Root D.E., Doench J.G. (2014). Genome-scale CRISPR-Cas9 knockout screening in human cells. Science.

